# Brain expression profiles of two *SCN1A* antisense RNAs in children and adolescents with epilepsy

**DOI:** 10.1515/tnsci-2022-0330

**Published:** 2024-01-23

**Authors:** Marius Frederik Schneider, Miriam Vogt, Johanna Scheuermann, Veronika Müller, Antje H. L. Fischer-Hentrich, Thomas Kremer, Sebastian Lugert, Friedrich Metzger, Manfred Kudernatsch, Gerhard Kluger, Till Hartlieb, Soheyl Noachtar, Christian Vollmar, Mathias Kunz, Jörg Christian Tonn, Roland Coras, Ingmar Blümcke, Claudia Pace, Florian Heinen, Christoph Klein, Heidrun Potschka, Ingo Borggraefe

**Affiliations:** Division of Molecular Biology, Biomedical Center Munich, Ludwig Maximilians University, Munich, Germany; International Max Planck Research School (IMPRS) for Molecular Life Sciences, Planegg-Martinsried, Germany; ISAR Bioscience GmbH, Planegg, Germany; Munich Medical Research School, Ludwig Maximilians University, Munich, Germany; Roche Pharmaceutical Research and Early Development, Roche Innovation Center Basel, F Hoffmann-La Roche Ltd, Basel, Switzerland; Versameb AG, Hochbergerstrasse 60C, 4057, Basel, Switzerland; Clinic for Neurosurgery, Schoen-Klinik Vogtareuth, Germany; Paracelsus Medical University, Salzburg, Austria; Neuropediatric Clinic and Clinic for Neurorehabilitation, Epilepsy Center for Children and Adolescents, Schoen-Klinik Vogtareuth, Germany; Department of Neurology, Comprehensive Epilepsy Center, University Hospital of Munich, Ludwig Maximilians University, Munich, Germany; Comprehensive Epilepsy Center, Division of Pediatric Neurology, Developmental Medicine and Social Pediatrics, Department of Pediatrics, University Hospital of Munich, Ludwig Maximilians University, Munich, Germany; Department of Neurosurgery, University Hospital of Munich, Ludwig Maximilians University, Munich, Germany; Department of Neuropathology, University Hospital Erlangen, Erlangen, Germany; Institute of Pharmacology, Toxicology, and Pharmacy, Ludwig Maximilians University, Munich, Germany; Division of Pediatric Neurology, Developmental Medicine and Social Pediatrics, Department of Pediatrics, University Hospital of Munich, Ludwig Maximilians University, Munich, Germany; Department of Pediatrics, University Hospital of Munich, Ludwig Maximilians University, Munich, Germany

**Keywords:** Dravet syndrome, long non coding RNA, regulatory RNA, precision medicine, epilepsy

## Abstract

**Objective:**

Heterozygous mutations within the voltage-gated sodium channel α subunit (*SCN1A*) are responsible for the majority of cases of Dravet syndrome (DS), a severe developmental and epileptic encephalopathy. Development of novel therapeutic approaches is mandatory in order to directly target the molecular consequences of the genetic defect. The aim of the present study was to investigate whether cis-acting long non-coding RNAs (lncRNAs) of *SCN1A* are expressed in brain specimens of children and adolescent with epilepsy as these molecules comprise possible targets for precision-based therapy approaches.

**Methods:**

We investigated *SCN1A* mRNA expression and expression of two *SCN1A* related antisense RNAs in brain tissues in different age groups of pediatric non-Dravet patients who underwent surgery for drug resistant epilepsy. The effect of different antisense oligonucleotides (ASOs) directed against *SCN1A* specific antisense RNAs on *SCN1A* expression was tested.

**Results:**

The *SCN1A* related antisense RNAs *SCN1A*-dsAS (downstream antisense, RefSeq identifier: NR_110598) and *SCN1A*-usAS (upstream AS, *SCN1A*-AS, RefSeq identifier: NR_110260) were widely expressed in the brain of pediatric patients. Expression patterns revealed a negative correlation of SCN1A-dsAS and a positive correlation of lncRNA *SCN1A*-usAS with *SCN1A* mRNA expression. Transfection of SK-N-AS cells with an ASO targeted against *SCN1A*-dsAS was associated with a significant enhancement of *SCN1A* mRNA expression and reduction in *SCN1A*-dsAS transcripts.

**Conclusion:**

These findings support the role of *SCN1A*-dsAS in the suppression of *SCN1A* mRNA generation. Considering the haploinsufficiency in genetic *SCN1A* related DS, *SCN1A*-dsAS is an interesting target candidate for the development of ASOs (AntagoNATs) based precision medicine therapeutic approaches aiming to enhance *SCN1A* expression in DS.

## Abbreviations

ASM: antiseizure medication
ASO: antisense oligonucleotide
dsAS: downstream antisense
DS: Dravet syndrome
FCD: focal cortical dysplasia
GG: ganglioglioma
HS: hippocampal sclerosis
KD: ketogenic diet
LGT: low grade tumor
lncRNA: long non-coding RNA
MCD: malformation of cortical development
MOGHE: mild malformation of cortical development with oligodendroglial hyperplasia
mMCD: mild malformation of cortical development with heterotopic neurons in the white matter
MVNT: multinodular and vacuolating neuronal tumor
PMG: polymicrogyria
SCN1A: sodium channel 1 α gene
TPB: TATA-Box binding protein
usAS: upstream antisense

## 1 Introduction

Dravet syndrome (DS) is a severe developmental and epileptic encephalopathy (DEE) with manifestation in early infancy. The incidence in the US is estimated to be 1/150,000 [1]. In the majority of cases, DS is caused by heterozygous loss-of-function mutations within the voltage-gated sodium channel α subunit (*SCN1A*), which leads to haploinsufficiency of the type I voltage-gated sodium channel Na_V_1.1 [2]. The latter is primarily expressed on axons of fast spiking GABAergic inhibitory interneurons. The *SCN1A* mutations result in impaired synaptic Na^+^ currents causing an impairment of GABAergic inhibition of downstream neurons [3,4]. The clinical hallmarks of *SCN1A* related DS are multiple seizure types, cognitive deterioration, behavioral disturbances, and ataxia [5]. Furthermore, the risk of sudden unexpected death in epilepsy is about 15-fold higher in DS compared to other epilepsies [6,7]. Although most patients receive polytherapy with antiseizure medications, only a minority of patients will become seizure free. In addition, relief of cognitive, behavioral, and motor disturbances is usually not achieved by treatment with current available Antiseizure medication (ASMs). Eventually, epilepsy specific parameters (i.e., as frequency of status epilepticus) seem to have only a minor predictive value for severity of cognitive decline [8,9]. Thus, future treatment approaches should go beyond mere seizure suppression and aim to ameliorate the entire spectrum of symptoms in DS patients. In this context, the development of approaches directly targeting the molecular consequences of the genetic *SCN1A* defect are of particular interest. In DS patients with *SCN1A* deficiency, the haploinsufficiency opens opportunities for molecular targeting approaches aiming to upregulate *SCN1A.*

**Figure 1:**
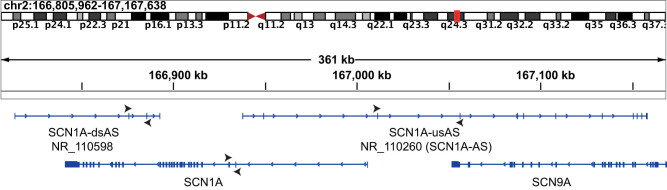
Human locus of *SCN1A* and its antisense transcripts *SCN1A*-dsAS (NR_110598) and *SCN1A*-usAS (NR_110260) located downstream to SCN9A on chromosome 2 [11]. Black arrows around transcripts indicate primer location (top: forward primer, bottom: reverse primer).

Long non-coding RNAs (lncRNAs) have been identified as a novel subcategory of regulatory RNA molecules. Especially, lncRNAs, oriented in the opposite direction to protein-coding genes and defined as antisense RNAs, exert a pronounced effect on gene expression with a specific impact on the neighboring gene [10]. In humans, the *SCN1A* transcription locus is surrounded by gene sequences encoding two antisense RNAs (Figure 1). One transcript with the RefSeq identifier NR_110598 is located downstream to SCN1A, and therefore further defined as *SCN1A* downstream AS (*SCN1A*-dsAS). The other transcript with the identifier NR_110260 is located upstream to *SCN1A* and downstream to *SCN9A* and referred to as *SCN1A* upstream AS (*SCN1A*-usAS, Figure 1).

Recently, it was shown that expression of *SCN1A* could be enhanced by targeting *SCN1A*-dsAS with antisense oligonucleotides (ASOs) on both mRNA and protein level [12]. Therefore, *SCN1A-*dsAS appears to be an interesting transcript as a target for precision medicine approaches in *SCN1A* related DS. However, further development of this approach for neuropediatric patients requires information about the expression of this potential target antisense RNA during human brain development and across the age range, and about its regional distribution in different brain areas.

In the present study, we therefore investigated the expression of *SCN1A* related antisense RNAs in surgical brain tissue specimen from pediatric patients. The ratio between antisense RNAs and *SCN1A* mRNA expression rates were analyzed in order to gain knowledge about the regulatory impact of antisense RNAs *SCN1A*-dsAS and -usAS on SCN1A expression.

## 2 Methods

### 2.1 Patient selection

Patients were selected from two tertiary epilepsy centers (Munich and Vogtareuth, Germany). All patients suffered from medically refractory epilepsy defined as resistant to at least two common ASMs appropriate for the underlying epilepsy syndrome. All included patients underwent a thorough presurgical diagnostic evaluation including a long-term EEG/video monitoring and high-resolution MRI as reported previously [13]. The decision for surgery was taken in an interdisciplinary case conference involving neurologists, pediatric neurologists, neurosurgeons, neuropsychologists, and neurophysiologists.

### 2.2 Specimen collection

Three to four biopsy specimens (maximum about 5 × 5 × 5 mm/biopsy) per patient were collected and immediately frozen on dry ice. Specimens were either directly transferred for further processing to the laboratory (Munich) or stored at −70°C for further transport and processing (Vogtareuth). A detailed protocol including documentation of all steps was applied for all specimens in order to ensure an accurate way of transportation and continuous cooling.

### 2.3 Histopathological evaluation

Routine histological evaluation of brain specimens was performed by the Neuropathological Reference Center for Epilepsy surgery of the University of Erlangen.

### 2.4 RNA-isolation

100 mg tissue was grounded in 900 µL of Isol-RNA Lysis Reagent (VWR, Germany) and incubated at room temperature for 10 min. The suspension was cleared at 17.000 g and 4°C for 5 min and treated with 200 µL chloroform (Roth, Germany) for 5 min under continuous shaking. The aqueous phase was separated from the organic phase by spinning at 17.000*g* and 4°C for 20 min. RNA was precipitated from the aqueous phase with 650 µL isopropanol (Roth) at −20°C for at least 1 h. RNA was pelleted at 17.000*g* and 4°C for 35 min and then washed twice with 75% ethanol (Roth). The RNA pellet was air-dried and dissolved in 15–35 µL nuclease-free water. For re-purification, RNA was treated with 350 µL of lysis buffer (Qiagen, Germany) and 100 µL ethanol and loaded on RNAeasy^®^ spin column (Qiagen, Germany). Further purification was performed according to the manufacturer’s instructions. RNA quality was assessed on an analytical agarose gel or with the qubitTM RNA IQ assay kit (Thermo Fischer Scientific, MA, USA).

### 2.5 First-strand cDNA synthesis (reverse transcription)

0.375 µg Oligo-dT12-18 (Thermo Fischer Scientific, MA, USA) were annealed to 500–1,000 ng bulk RNA together with 7.5 nmol of each dNTPs in a 9 µL reaction scale. Thereafter, the RNA was first denatured at 65°C for 5 min and subsequently cooled down to 37°C. Afterwards, 3 µL 5× First-strand buffer (Thermo Fischer Scientific, MA, USA), 1.5 µL DTT (100 mM, Thermo Fischer Scientific, MA, USA), 30 U murine RNAse Inhibitor (New England Biolabs, USA), and 150 U M-MLV reverse transcriptase (Thermo Fischer Scientific, MA, USA) were added to the reaction. After 45 min of incubation at 37°C, the reaction was stopped at 70°C for 10 min. For expression analysis by qPCR, the cDNA was diluted with 185 µL of nuclease-free water.

### 2.6 Quantitative RT-PCR analysis

2 µL of cDNA was amplified in a 10 µL 1× Fast SYBR^®^ Green Master Mix (Thermo Fischer Scientific, MA, USA) in the presence of 2.5 pmol of each primer. Three technical replicates were run per primer pair. The reaction was run in an Applied Biosystems StepOne^TM^ or Biorad CFX-384 cycler with the following settings: 95°C, 3 min for initial denaturing and 40 cycles with 95°C, 3 s and 60°C, 30 s. Typically, we designed primers annealing either to two subsequent exons or at least to the border between both exons to avoid the amplification of genomic DNA (Table 1). For normalization and quantification using the ∆Ct method, TBP served as a housekeeping gene. Two to four biopsy specimens per patient were analyzed to calculate the mean expression 2^(ΔCt)^ value.

**Table 1:** Primer sequence information for *SCN1A*, *SCN1A*-dsAS, and *SCN1A*-usAS

Target	Primer	5′→ 3′ sequence
TBP	TBP-fw	CGGTTTGCTGCGGTAATCAT
	TBP-rv	GGTGAGCACAAGGCCTTCTA
SCN1A	SCN1A-fw	GCTGTATACATCCGTGTGCAG
	SCN1A-rv	GCGTCTTTCAATAGCCGCAA
SCN1A-dsAS	SCN1A-dsAS-fw	CCTTGTACACCAGGGCATTC
	SCN1A-dsAS-rv	GTGGTATAGGAACTGGCAGCA
SCN1A-usAS	SCN1A-usAS-fw	CCAGGAAACAGGAATTCAGGC
	SCN1A-usAS-rv	CGAGTGATCCGTCTTGCCA

fw = forward, rv = reverse, TBP (housekeeping) = TATA box binding protein, SCN1A = sodium channel 1 α subunit, SCN1A-dsAS = SCN1A downstream antisense transcript: NR_110598, SCN1A-usAS = SCN1A upstream antisense transcript: NR_110260.

### 2.7 Statistical analysis

The correlation of *SCN1A* and its antisense transcripts were evaluated with R 3.6 using the Pearson correlation method to calculate *R* ^2^ and *p*-value. The student’s *t*-test for unpaired samples was applied to evaluate intergroup differences. The level of significance level was set to 0.05 (two-tailed). In case of multiple testing, the significance level was adjusted applying the correction method of Bonferroni [14].

### 2.8 ASOs directed against *SCN1A*-dsAS

ASO specifications are depicted in Table 2.

**Table 2:** Detailed specifications of ASO. ASO2 is not shown as it was not ordered due to predicted inappropriate binding properties

ASO	Modified seg: *phosphorothioates, 2MoEr: 2'methodxyethoxy moiety	Scale (nmol)
scr-ctrl	/52MOErC/*/i2MOErT/*/i2MOErA/*/i2MOErA/*/i2MOErG/*G*T*T*A*A*G*T*C*G*C*/i2MOErC/*/i2MOErC/*/i2MOErT/*/i2MOErC/*/32MOErG/	100
ASO1 (nr110598-1)	/52MOErC/*/i2MOErA/*/i2MOErC/*/i2MOErG/*/i2MOErG/*A*A*G*A*C*T*T*T*A*G*/i2MOErT/*/i2MOErA/*/i2MOErG/*/i2MOErT/*/32MOErG/	100
ASO3 (nr110598-3	/52MOErG/*/i2MOErG/*/i2MOErT/*/i2MOErA/*/i2MOErT/*A*G*G*A*A*C*T*G*G*C*/i2MOErA/*/i2MOErG/*/i2MOErC/*/i2MOErA/*/32MOErG/	100

### 2.9 Cell culture and transfection of cell lines

SK-N-AS cells (human neuroblastoma derived cell line) were cultured for 48 h (5% CO_2_) at 37°C. 40pmol of ASO were transfected with Lipofectamin2000 (Thermo Fisher Scientific, Waltham, MA, USA) according to the manufactures’ protocol. RT-PCR of *SCN1A* and *SCN1A*-dsAS was performed 48 h after transfection as described in Section 2.6.


**Ethical approval:** The research related to human use has been complied with all the relevant national regulations, institutional policies and in accordance with the tenets of the Helsinki Declaration, and has been approved by the authors’ institutional review board or equivalent committee. The responsible ethical boards (Munich: ethical board of the Medical Faculty of the University of Munich, Vogtareuth: ethical board of the Bavarian professional association of physicians) approved the study (# 18-331 and # 18042, respectively). Patient data after study entry were pseudonymized.
**Informed consent:** Informed consent has been obtained from all individuals included in this study.

## 3 Results

### 3.1 Demographic data

We investigated the brain specimens of a total of 18 patients (for summary of demographic data, see Table 3, for a detailed individual case description, see Table 4) of whom 14 were females (77.8%). The mean age at the time of surgery was 9.1 years (±5.1, range 0.8–18.7) and the mean duration of epilepsy was 6.5 years (±4.5, range 0.8–15.7). Most of the patients suffered from daily seizures at the time of surgery (*n* = 12, 66.7%). Only a minority of the patients had seizures on a weekly or monthly basis (*n* = 3, 16.7% and *n* = 3, 16.6%). The regional distribution of specimen origin was as follows: frontal lobe 50% (*n* = 10), temporal lobe 40% (*n* = 8), parietal lobe 5% (*n* = 1), and insula 5% (*n* = 1).

**Table 3:** Summary of demographic patient data

Total (*n* =)	18
Sex (f/m)	14/4
Mean age at surgery (years)	9.1 (±5.1, range 0.8–18.7)
Mean age at seizure manifestation (years)	2.6 (±2.5, range 0.1–8.0)
Mean epilepsy duration (years)	6.5 (±4.5, range 0.8–15.7)
**Seizure frequency (** ***n*** **=)**	
Daily	12
Weekly	3
Monthly	3
Number of previous ASMs (mean value)	5 (±3, range 1–12)
Previous KD (*n* =)	2
Number of ASMs at surgery (mean value)	2 (±1, range 1–4)
**Resected tissue area (** ***n*** **=)**	
Frontal	10
Temporal	8
Parietal	1
Insular	1
**Pathology (** ***n*** **=)**	
MCD	13
HS	3
LGT	2
Gliosis	3

ASMs = antiseizure medications, KD = ketogenic diet, MCD = malformation of cortical development, HS = hippocampal sclerosis, LGT = low grade tumor.

**Table 4:** Detailed summary of individual patient specifications

Patient ID	Sex	Age at surgery (years)	Seizure manifestation (years)	Epilepsy duration (years)	Seizure frequency	Number of previous ASMs	ASMs at surgery	Type of surgery/resection	Investigated tissue	Pathology
SKV-13	M	13.3	0.3	12.9	Daily	2	1	Temporal lobe resection, amygdalohippocampectomy, anterior insular, frontal opercular, and basal cortex	Anterior temporal lobe	HS, FCD IIIa
SKV-04	W	4.2	0.2	4.0	Daily	12	2	Subtotal frontal and anterior insular resection	Frontal lobe	MOGHE
SKV-03	M	4.8	1.4	3.4	Daily	10	2	Subtotal frontal and anterior insular and anterior cingular resection	Frontal lobe	MOGHE
SKV-05	M	11.5	8.0	3.5	Daily	1	1	Temporal lobe resection	Anterior temporal lobe	MVNT
SKV-08	W	8.5	5.0	3.5	Weekly	2	1	Temporal lobe resection	Anterior temporal lobe	GG
LMU-16	W	6.5	5.0	1.5	Weekly	3	1	Parietal lesionectomy	Parietal lobe	FCD Ia
LMU-17	W	0.8	0.1	0.8	Daily	4	4	Hemispherotomy	Frontal and parietal lobe, insula	FCD IIb, PMG
SKV-01	W	1.3	0.3	1.0	Weekly	7	4	Hemispherotomy	Frontal lobe	FCD IIa
SKV-06	W	7.3	2.0	5.3	Daily	5	2	Hemispherotomy	Frontal lobe	Gliosis
SKV-07	M	13.2	0.5	12.7	Daily	4	2	Hemispherotomy	Frontal lobe	Gliosis
SKV-10	W	7.3	1.4	5.9	Daily	4	4	Subtotal frontal lobe resection	Frontal lobe	MOGHE
SKV-14	W	7.7	2.0	5.7	Monthly	4	2	Temporal lobe resection	Anterior temporal lobe	HS
SKV-15	W	18.7	3.0	15.7	Daily	3	2	Temporal lobe resection	Anterior temporal lobe	HS
SKV-12	W	12.1	3.5	8.6	Daily	4	2	Hemispherotomy	Frontal lobe	PMG
SKV-02	W	12.2	0.6	11.6	Daily	10	4	Hemispherotomy	Frontal lobe	FCD IIId, gliosis
SKV-09	W	13.9	7.2	6.8	Monthly	5	2	Temporal lobe resection	Temporal lobe	mMCD
SVK-11	W	16.1	5.5	10.6	Month	3	2	Temporal lobe resection	Anterior temporal lobe	FCD IIa
LMU-18	W	4.0	0.6	3.4	Daily	6	2	Hemispherotomy	Frontal lobe	MOGHE

Note: Patients SVK-03 and 13 received ketogenic diet as a presurgical antiseizure treatment. Abbreviations: HS = hippocampal sclerosis, FCD = focal cortical dysplasia, GG = ganglioglioma, MOGHE = mild malformation of cortical development with oligodendroglial hyperplasia, MVNT = multinodular and vacuolating neuronal tumor, mMCD = mild malformation of cortical development with heterotopic neurons in the white matter, PMG = polymicrogyria, ASMs = antiseizure medications.

### 3.2 *SCN1A* and *SCN1A*-lnc expression and correlation

In the first extraction step, 51.8 ± 24.9 µg total RNA was purified from 100 mg tissue. After the second column purification step, we obtained 63.26 ± 37.9% of the used RNA with an RNA-IQ value of 8.2 ± 0.4, showing that the purification procedure yielded RNA with high quality. Next we measured the level of *SCN1A*, *SCN1A*-dsAS, and *SCN1A*-usAS with quantitative RT-PCR using TBP as a housekeeping gene [15].

**Figure 2:**
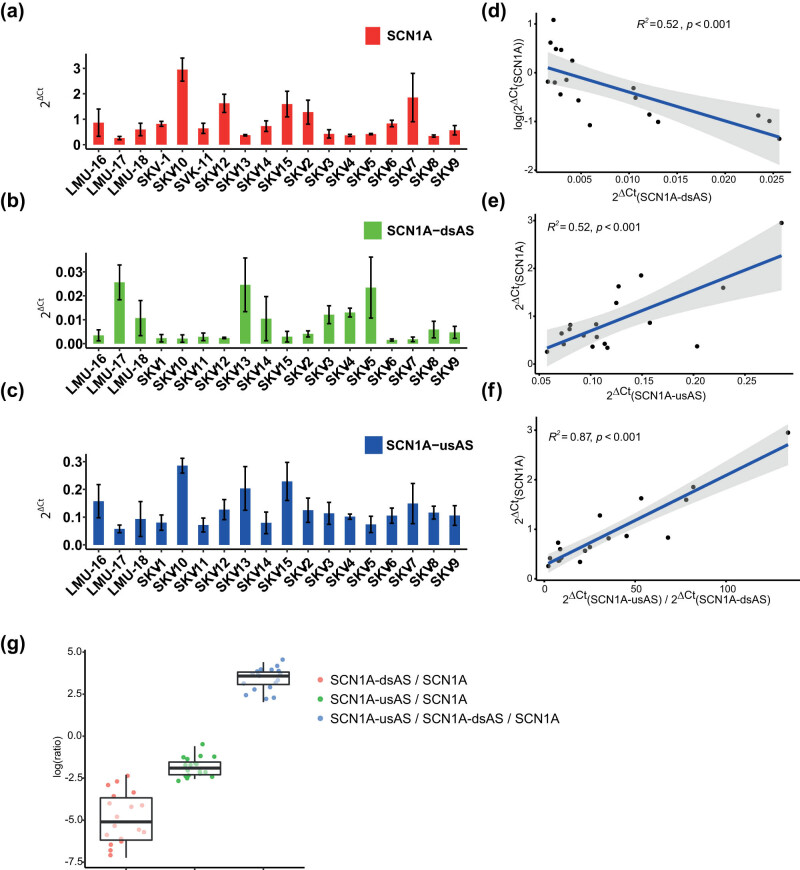
*SCN1A* expression correlates with the expression of *SCN1A*-dsAS and *SCN1A*-usAS. (a–c) RNA expression levels of *SNC1A* (a), *SCN1A*-dsAS (b), and *SCN1A*-usAS (c) determined by quantitative RT-PCR in biopsy specimens dissected from patients who underwent surgery for drug resistant epilepsy. Values are normalized to TBP. Expression values were measured in 2–4 biopsy specimens per patient. (d–f) Correlation analysis of the expression of *SCN1A* and its antisense transcripts. The correlation coefficient is provided as *R* ^2^-value in the plot. (d) Correlation of *SCN1A* expression levels with *SCN1A*-dsAS expression levels (log scale). (e) Correlation of *SCN1A* expression levels with *SCN1A*-usAS expression levels. (f) Correlation of *SCN1A* expression levels with the ratio of *SCN1A*-usAS and *SCN1A*-dsAS expression levels. (g) Boxplot analysis showing distribution of: *SCN1A*-dsAS/*SCN1A* ratio (red), *SCN1A*-usAS/*SCN1A* ratio (green), and *SCN1A*-usAS/*SCN1A*-dsAS/*SCN1A* ratio (blue, log scale).

*SCN1A* was the most abundant transcript (Figure 2a). Among the regulatory antisense RNAs, *SCN1A*-dsAS was the least abundant antisense transcript (Figure 2b), and *SCN1A-*usAS exhibited a moderate expression (Figure 2c). The ratio between the individual expression of *SCN1A*, *SCN1A-*dsAS, and *SCN1A*-usAS was comparable among patients (Figure 2g).

Next we completed a correlation analysis to assess a putative regulatory impact of the antisense transcripts on *SCN1A* expression by correlation analysis. First, we analyzed a potential correlation of *SCN1A’*s with *SCN1A*-dsAS’s expression. This analysis demonstrated a significant negative correlation (*R* ^2^ = 0.5, *p* < 0.0001, Figure 2d). In contrast, the respective analysis for *SCN1A* and *SCNA1*-usAS’s expression revealed a positive correlation (*R* ^2^ = 0.52, *p* < 0.0001, Figure 2e).

To test whether both transcripts co-regulate *SCN1A*’s expression, we additionally investigated the correlation of *SCN1A* expression with the expression ratio of both antisense transcripts. A strong correlation became evident between *SCN1A* expression and the expression ratio of the antisense transcripts (*R* ^2^ = 0.87, *p* < 0.0001, Figure 2f). An assessment of a potential direct correlation between the regulatory RNAs did not confirm a significant correlation (*R* ^2^-value = 0.0625; *p* = 0.32, no plot shown).

**Figure 3:**
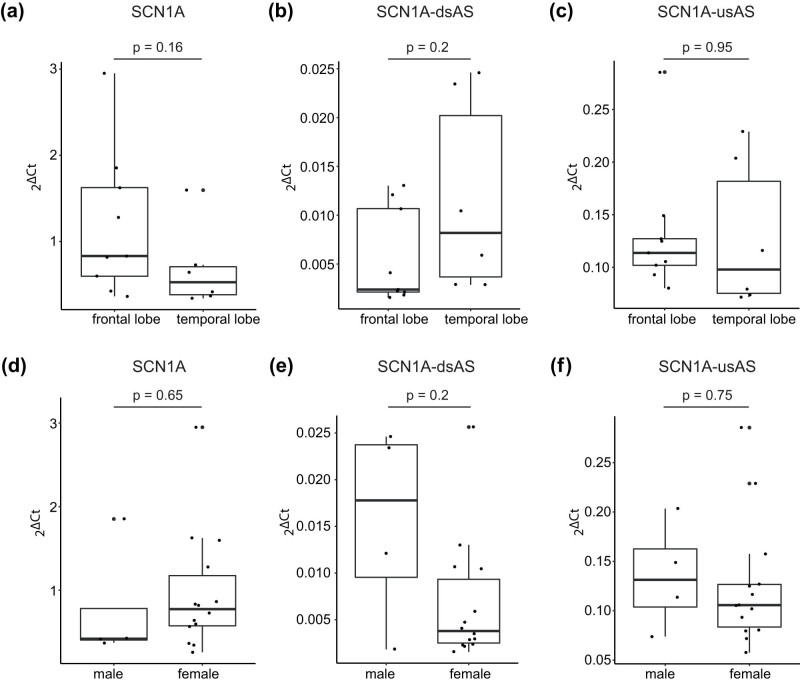
*SCN1A*, *SCN1A*-dsAS, and *SCN1A*-usAS are equally expressed in frontal and temporal lobes and in both sexes. (a–c) Boxplots show the expression of *SCN1A* (a), *SCN1A*-dsAS (b), and *SCN1A*-usAS (c) in the frontal lobe and temporal lobe. (d–f) Boxplots show the expression of *SCN1A* (d), *SCN1A*-dsAS (e), and *SCN1A*-usAS (f) in the male and female patients.

**Figure 4:**
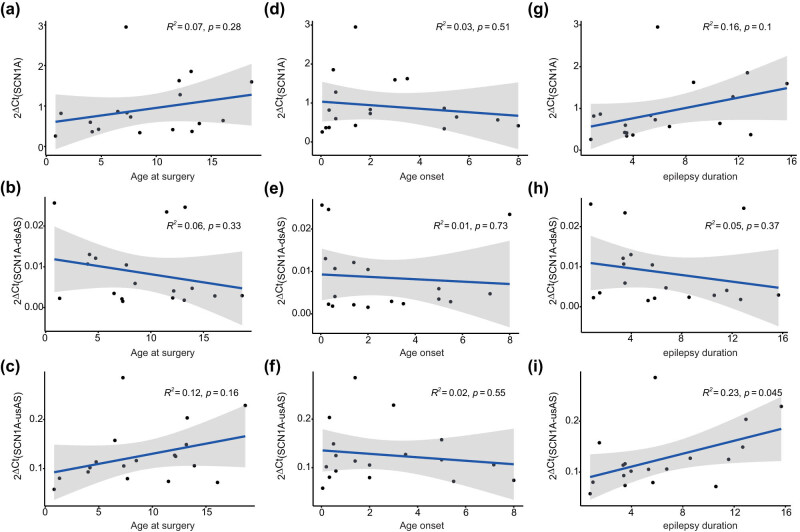
Age at surgery, age of disease onset, and duration of epilepsy do not correlate with *SCN1A*, *SCN1A*-dsAS, and *SCN1A*-usAS expression. (a–c) Correlation of *SCN1A* expression (a), *SCN1A*-dsAS (b), and *SCN1A*-usAS (c) for age at surgery. (d–f) Correlation of *SCN1A* expression (d), *SCN1A*-dsAS (e), and *SCN1A*-usAS (f) for age of onset. (g–i) Correlation of *SCN1A* expression (g), *SCN1A*-dsAS (h), and *SCN1A*-usAS (i) for duration of epilepsy.

We detected no difference in the expression patterns of *SCN1A, SCN1A*-dsAS, and *SCN1A-*usAS with respect to lobar location (Figure 3a–c) and sex (Figure 3d–f). Furthermore, there was no correlation of either transcript expression for age at surgery (Figure 4a–c), age of epilepsy onset (Figure 4d–f) or epilepsy duration (Figure 4g–i).

### 3.3 Treatment of SK-N-AS cells with ASOs directed against *SCN1A*-dsAS

**Figure 5:**
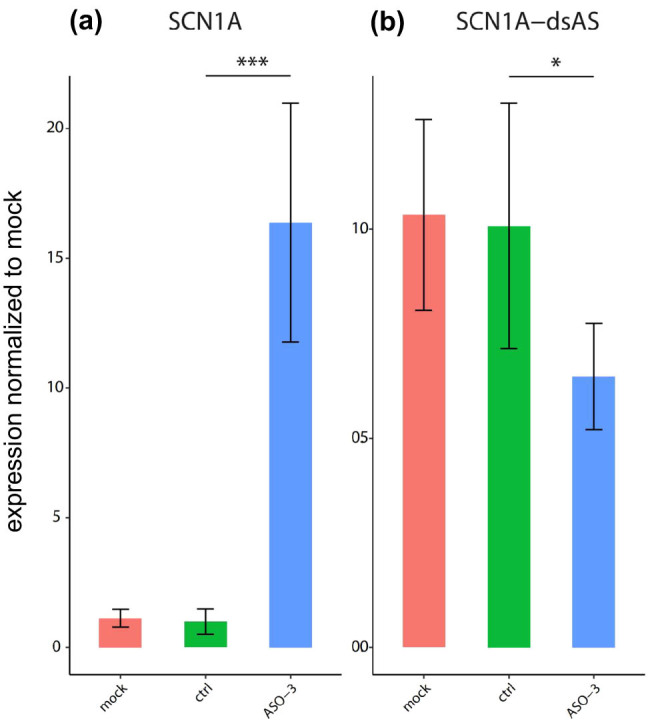
Transfection of SK-N-AS cells with ASOs directed against *SCN1A*-dsAS. Treatment with ASO3 showed a significant increase in *SCN1A* mRNA expression by about 15-fold (a, ***p < 0.001) and reduction in *SCN1A*-dsAS expression by about 40% compared to control and mock transfected cells (b, *p < 0.05).

Three ASOs targeted against *SCN1A-*dsAS were designed. Treatment with ASO3 revealed a significant increase (*p* < 0.001) in *SCN1A* mRNA expression by about 15-fold compared to control or mock transfected cells (Figure 5a). Furthermore, ASO3 treatment was associated with a reduction in *SCN1A*-dsAS expression by about 40% (*p* < 0.05) compared to control and mock transfected cells (Figure 5b). ASO1 did not alter the expression of either *SCN1A* mRNA or *SCN1A*-dsAS transcript (not shown).

## 4 Discussion

In the present study, we analyzed RNA-expression of *SCN1A* and two *SCN1A* antisense RNAs (*SCN1A-*dsAS and *SCN1A*-usAS) in brain specimens of children and adolescents undergoing surgery for medically refractory epilepsy. *SCN1A*-dsAS correlated negatively and *SCN1A*-usAS positively with *SCN1A* expression suggesting a contrary role in *SCN1A* gene regulation for both antisense RNAs.

*SCN1A* related DS is a devastating DEE. There are recent developments of new antiseizure medications as fenfluramine and cannabidiol. These drugs can significantly reduce seizure frequency compared to placebo though seizure freedom is only rarely achieved [16,17]. However, the impact of “newer” and “older” antiseizure medications on further clinically relevant symptoms in DS patients including cognitive decline, behavioral disturbances, and atactic gait disorder seems to be rather limited. Thus, there is an urgent need for disease-modifying agents overcoming Na_V_1.1 haploinsufficiency. This need is underlined by both clinical and experimental findings that seizure activity alone is only a poor contributor to cognitive deterioration [9,18]. In the sense of precision medicine, various options are currently under investigation. Among others, these include the application of selective Na_v_1.1 activators, CRISPRa/dCAS technologies targeting transcription, of ASOs to target either mRNA processing or, as antagonists of cis-acting antisense RNAs [19–22]. These approaches aim to either ameliorate the haploinsufficiency of Na_v_1.1 by enhancing the activity or by upregulating the expression of residual Na_v_1.1. The inhibition of antisense RNAs on *SCN1A* has been recently extensively investigated *in vitro* and *in vivo* [12]. The latter study by Hsiao and colleagues revealed that *SCN1A* expression can be enhanced by application of ASOs against *SCN1A* related antisense RNAs in several cell lines, fibroblasts of patients with DS, and brain tissue of both mice and monkeys [12]. Moreover, seizure activity was ameliorated by this approach in mice carrying a pathogenic heterozygous *SCN1A* mutation.

### 4.1 Expression of *SCN1A* and *SCN1A*-dsAS/*SCN1A*-usAS in human brain tissue

*SCN1A* and *SCN1A*-dsAS/*SCN1A*-usAS proved to be expressed in the investigated brain regions primarily comprising the frontal and temporal lobe. According to the human protein atlas (brain-map.org) and previous reports, *SCN1A* is known to be widely expressed in these areas [23,24]. The expression in these brain regions is thought to be connected to the key clinical features in DS as cognitive and behavioral disturbances (frontal lobe) and seizures (temporal lobe, hippocampus) [18,25]. We noticed that the expression of all three transcripts varied significantly between the patients. However, the ratio of the individual expression of *SCN1A*, *SCN1A*-dsAS, and *SCN1A*-usAS was comparable among patients. These results suggest similar regulatory mechanisms between the transcripts despite interindividual differences in quantitative expression levels. There was no correlation between transcript and the age of investigation (= age at surgery). These findings support that potential targets for treatment, i.e., with ASOs against *SCN1A*-dsAS are detectable within the time span of the investigated patients from 1 to 19 years of age. This finding is of particular relevance as any treatment option aiming to modify the disease should be most likely implemented as early as possible in order to overcome long term sequelae of concurrent Na_v_1.1 dysfunction and ongoing seizure activity on the developing brain [26–28].

Epilepsy duration had no impact on regulation of *SCN1A*, *SCN1A*-dsAS, and *SCN1A*-usAS (Figure 4g–i). As epilepsy duration also reflects the total load of seizures until surgery, the findings may suggest that seizure activity does not affect *SCN1A*, *SCN1A-*dsAS, and *SCN1A*-usAS expression in brain tissues derived from non-Dravet patients. Our data on expression of *SCN1A*-dsAS in human brain tissues complement findings of previous studies reporting *SCN1A* antisense RNAs expression in mouse and monkey brain tissue [12]. In the latter study, the researchers also detected *SCN1A* antisense RNAs expression in brain tissue derived from a human tissue panel.

### 4.2 Correlation of expression levels of *SCN1A* and *SCN1A-*dsAS/*SCN1A*-usAS in human brain tissue

*SCN1A* expression correlated in an opposite manner with the two *SCN1A* related antisense RNAs: *SCN1A*-dsAS correlated negatively and *SCN1A*-usAS positively with *SCN1A* expression on mRNA level. The data suggest that both antisense transcripts co-modulate *SCN1A*’s expression without affecting the expression of the other antisense transcript. While *SCN1A*-dsAS most likely suppresses SCN1A expression, *SCN1A*-usAS may enhance *SCN1A* expression. Thus, these results support that inhibition of *SCN1A*-dsAS, i.e., by ASOs might help to increase *SCN1A* gene expression. As mentioned above, functional evidence has been reported that *SCN1A* gene expression on mRNA and protein level *in vitro* is enhanced by ASOs against *SCN1A-*dsAS RNA and improves *in vivo* seizure activity in a Dravet mouse model [12]. Contrary to our results in human tissue, the latter study reported a positive correlation of the downstream antisense RNA and *SCN1A* in primate (green monkey) tissue samples. They interpreted these findings as an increased turnover of antisense RNAs in more “active” loci. However, the results are difficult to compare with our findings as they used mixed tissue samples consisting of more than only brain tissue. Furthermore, potential species differences might be applicable in this context.

As DS is commonly caused by haploinsufficiency of truncating or less commonly missense mutations of the *SCN1A* gene, there is no dominant negative effect of mutations [29]. Thus, it is rather unlikely that upregulation of *SCN1A* will exert detrimental effects in the affected neurons. The unlikelihood of dominant negative effects further underlines the potential value of ASOs against *SCN1A*-dsAS as a precise and disease modifying therapeutic concept for patients with DS. This is further underlined by current approaches and ongoing phase 1/2 studies to enhance *SCN1A* expression by overcoming introduction of the poison exon 20 N insertion within the *SCN1A* transcript [20,30]. Insertion of exon 20 N into a *SCN1A* transcript is known to reduce functioning Na_v_1.1 channel expression. Nevertheless, recent findings have reported on patients with *SCN1A* gain of function mutations [31]. In these patients, enhancement of *SCN1A* mRNA expression is not warranted as worsening of the condition is very likely.

In addition to SCN1A-dsAS, SCN1A-usAS, whose expression positively correlates with SCN1A expression, could also be a suitable target for increasing SCN1A expression. Similar to the reported cases in which lncRNAs such as PCNA-AS enhance the expression of nearby protein-coding genes by repressing miRNAs targeting these genes, there is a possibility that a similar regulatory network exists between SCN1A-usAS and SCN1A [32]. If this regulatory link is confirmed, exogenous expression of SCN1A-usAS to enhance SCN1A expression could be exploited using recombinant adeno-associated viruses that do not deliver 13kbp targets like SCN1A [33].

### 4.3 Treatment of SK-N-AS cells with ASOs directed against *SCN1A*-dsAS

Treatment with ASO3 targeted against *SCN1A*-dsAS revealed a significant increase in *SCN1A* mRNA expression and a reduced abundance of *SCN1A*-dsAS transcript in SK-N-AS cells. In our study, ASO3 demonstrated superior efficiency when compared to a previously published ASO (Cur-1740, 11), despite both targeting similar sites within the *SCN1A*-dsAS sequence. This enhanced efficacy could be attributed to distinct chemical modifications employed in our ASO design, specifically the incorporation of 2′O-methoxy-ethyl as opposed to alternative 2′O modification methods. This choice of chemical modification is known to elevate the melting temperature of the ASO and enhance its overall affinity towards the target gene [34]. The increased stability conferred by the 2′O-methoxy-ethyl modification potentially contributes to a more robust and effective binding of the ASO3 towards *SCN1A*-dsAS, resulting in a higher overall efficiency in upregulation of *SCN1A*

These data further underline the role of *SCN1A*-dsAS in regulation and especially in inhibition of *SCN1A* mRNA expression as investigated previously [12]. Reduced *SCN1A*-dsAS transcripts in ASO3 transfected cells further suggest that the enhancement of *SCN1A* mRNA Expression is in part negatively correlated with the amount *SCN1A*-dsAS lncRNA.

### 4.4 Limitations

There are several limitations, which should be acknowledged when interpreting the results of the present study. Measuring gene expression is a descriptive rather than a functional approach. In addition, due to posttranscriptional and posttranslational regulation mRNA expression does not always correlate with the expression of a fully functional protein [35). Nevertheless, a correlation of *SCN1A* mRNA expression and protein function was demonstrated in recent studies [12,20). In addition, concern on interpreting only quantitative RNA levels does not apply to regulatory RNA levels including lncRNAs.

One needs further to consider the heterogeneity of the samples (i.e., different pathological findings) and of the patient specifications for interpretation of the findings. However, the aim of this study was to determine whether the regulatory antisense RNAs are expressed at a relevant level in the epileptic brain at different age levels. Therefore, the aim implies a respective heterogeneity. In this context, it also should be underlined that we did not investigate brain tissues from *SCN1A* positive patients as these do not qualify for epilepsy surgery as investigated previously [36,37]. Thus, investigating brain specimen from *SCN1A* patients appears to be more than challenging. Nevertheless, caution is warranted when comparing results from non-Dravet to DS patients. We cannot exclude that *SCN1A* antisense transcripts are differently regulated in the brains of *SCN1A*-haploinsufficient patients. However this is at least not true for mouse models [12].

Furthermore, the use of control brain tissue could be suggested. However, taking intact brain specimen of children for a scientific study is questionable from an ethical point of view. Thus, we decided against using normal tissue in this study.

It is also emphasized that the sample size did not allow a meaningful subgroup comparison. While the data did not point to differences related to lobar location and sex or to a correlation of expression data with age of onset, age at surgery, and epilepsy duration, we cannot exclude that a larger sample size would have revealed differences or an association. There were no significant changes in the expression of *SCN1A* and both of its antisense transcripts with respect to lobar location and sex. Furthermore, age of onset, age at surgery, and epilepsy duration did not correlate with expression levels of either transcript. These findings might not only suggest a rather homogenous sample size but also could be interpreted as a result of the small sample size.

### 4.5 Conclusion

The findings of the present study revealed that *SCN1A* antisense RNAs are widely expressed across different human brain regions and age range. Correlation analysis suggests inhibitory effects of *SCN1A*-dsAS and enhancing effects of *SCN1A*-usAS on *SCN1A* expression. The data support *SCN1A*-dsAS as an interesting target for development of precision medicine therapeutic approaches for *SCN1A* related DS aiming to enhance expression of functional Na_v_1.1 and to overcome haploinsufficiency.
